# FGFR4 polymorphic alleles modulate mitochondrial respiration: A novel target for somatostatin analog action in pituitary tumors

**DOI:** 10.18632/oncotarget.13843

**Published:** 2016-12-09

**Authors:** Shereen Ezzat, Ri Wang, Melania Pintilie, Sylvia L Asa

**Affiliations:** ^1^ Department of Medicine, The Endocrine Oncology Site Group, Princes Margaret Cancer Centre, and the Ontario Cancer Institute, University Health Network, Toronto, Ontario, Canada; ^2^ Department of Statistics, University of Waterloo, Toronto, Canada; ^3^ Department of Biostatistics, University of Toronto, Toronto, Canada; ^4^ Department of Pathology, University Health Network, Toronto, Canada

**Keywords:** FGFR4, FGFR4-R388, mitochondrial functions, somatostatin analogs, pituitary tumors

## Abstract

We reported that a single nucleotide polymorphism (SNP) at codon 388 of the fibroblast growth factor receptor 4 (FGFR4-Gly388Arg) can result in distinct proteins that alter pituitary cell growth and function. Here, we examined the differential properties of the available therapeutic somatostatin analogs, octreotide and pasireotide, in pituitary tumor cells expressing the different FGFR4 isoforms. Consistent with their enhanced growth properties, FGFR4-R388-expressing cells show higher mitochondrial STAT3 serine phosphorylation driving basal and maximal oxygen consumption rate (OCR) than pituitary cells expressing the more common FGFR4-G388 isoform. While both somatostatin analogs reduce the OCR in FGFR4-G388 cells, pasireotide was more effective in decreasing OCR in cells expressing the variant FGFR4-R388 isoform. Down-regulation of somatostatin receptor 5 (SSTR5) abrogated the effect of pasireotide, demonstrating its involvement in mediating this action. The effects on OCR were recapitulated by introducing a constitutively active serine STAT3 but not by a tyrosine-active mutant. Moreover, pharmacologic inhibition demonstrated the role for the phosphatase PP2A in mediating the dephosphorylation of STAT3-S727 by pasireotide. Our data indicate that FGFR4 polymorphic isoforms mediate signaling that yields mitochondrial therapeutic targets of relevance to the actions of different somatostatin analogs.

## INTRODUCTION

Meta-analysis shows that pituitary tumors are common, occurring in almost 20% of the population [1]; they represent about 10 to 15% of surgically resected intracranial tumors [2–4]. However, the mechanisms underlying the development of sporadic pituitary tumors that rarely involve mutations of classical oncogenes or tumor suppressor genes remain to be clarified [2, 3]. Germline genetic abnormalities associated with pituitary tumor pathogenesis include inactivating mutations of menin in patients with Multiple Endocrine Neoplasia type 1 [5, 6], loss-of-function mutations of the aryl hydrocarbon receptor-interacting protein (AIP) tumor suppressor gene in patients with familial isolated pituitary adenomas [7], and inactivating mutations the Protein kinase A type I regulatory subunit PRKAR1A [8] in patients with Carney complex, however these alterations have not been shown to mediate pituitary neoplastic growth in the more common sporadic neoplasms.

Fibroblast growth factors (FGFs) are well known to have mitogenic, chemotactic, and angiogenic activities in cells of mesodermal and neuroectodermal origin. FGF signaling plays a crucial role in pituitary development [9, 10]. FGF receptor 4 (FGFR4) is a member of a family of transmembrane receptors with ligand-induced tyrosine kinase activity which have been implicated in tumorigenesis. A single nucleotide polymorphism (SNP) at codon 388 of FGFR4 (Gly388Arg), which encodes an amino acid in the transmembrane domain of the FGFR4 gene, was reported to be associated with poor outcome in sarcoma [11], prostate [12], lung [13, 14], colon [15], and head and neck carcinomas [16], melanoma [17], and advanced breast cancer [18]. In addition, this SNP was associated with more aggressive clinical behavior and resistance to mTOR inhibition therapy in patients with pancreatic neuroendocrine tumors [19]. Furthermore, more recently we showed that the FGFR4-R388 allele is associated with tumor growth and responsiveness to dexamethasone in Cushing's disease [20].

Acromegaly is an endocrine disorder characterized by increased circulating insulin-like growth factor-1 (IGF-1) levels usually due to a GH-secreting pituitary adenoma, leading to significant morbidity and excess mortality [21, 22]. Normalization of GH and IGF-1 levels results in an improvement of comorbidities [23–25] and a reduction in mortality [26–28]. Therapeutic options for patients with acromegaly include surgical removal of the GH-secreting pituitary adenoma, radiation therapy, and medical therapy with somatostatin analogs (SSAs), dopamine agonists, and/or a GH-receptor antagonist. SSAs can be used as the initial therapy or as a second therapy after failure of surgical treatment to achieve cure. They produce their effects, reduction of cell proliferation and inhibition of hormone production, by binding and activating somatostatin receptors (SSTRs), mainly SSTR2 and 5, expressed on the cell surface. However, some acromegalic patients show resistance to SSAs with failure of IGF-1 normalization. The predictive factors of responsiveness to SSAs include patient age, tumor size, baseline IGF-I or GH levels, and the pathological variant of GH-secreting adenoma [29–31]. In addition, molecular studies have examined the role of SSTR expression levels [32–34], mutation or polymorphism in SSTR 5 gene [35, 36], and decreased sensitivity of SSTR proteins [37–39], however, these studies have not fully explained the mechanisms of resistance to SSAs in acromegalic patients.

Recently we reported that the FGFR4-R388 allele modulates GH levels and is associated with larger pituitary tumor size in patients with acromegaly. We also demonstrated that this SNP can change mitochondrial functions through STAT3 serine translocation to the mitochondria, resulting in enhanced cell proliferation and hormone production through phosphorylation of STAT3 tyrosine in GH-producing pituitary cells [40]. Therefore, we hypothesized that this SNP would lead to higher IGF-1 levels and alter response to SSAs when compared to the prototypic form of the gene. Here we examined the differential impacts of the two FGFR4 isotypes on the two clinically available SSAs, octreotide which has high affinity for SSTR 2, and pasireotide with high affinity for SSTR5.

## RESULTS

### The FGFR4-R388 polymorphic allele promotes resistance to octreotide

**Figure 1:**
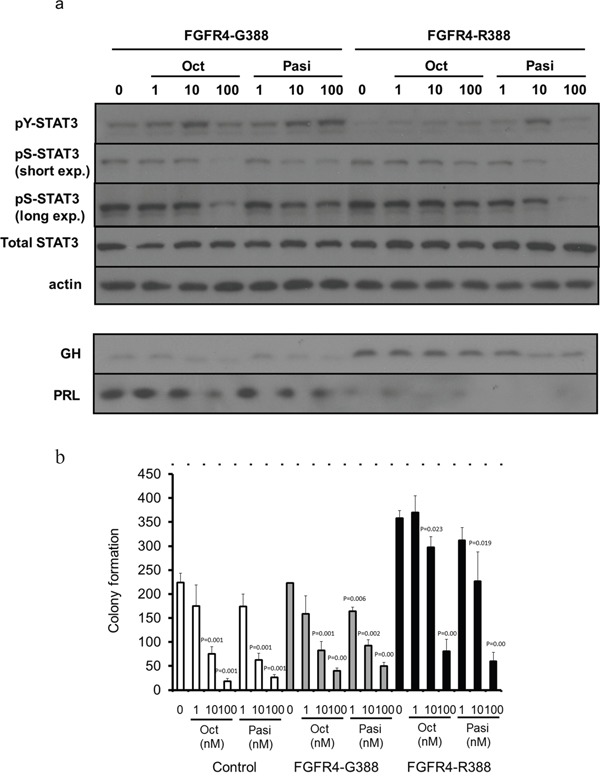
The FGFR4-R388 variant allele leads to resistance to octreotide but shows sensitivity to pasireotide in pituitary tumor cells **a**. GH4 cells expressing the common FGFR4-G388 or the variant FGFR4-R388 allele were treated with octreotide (Oct) or pasireotide (Pasi) under serum-free defined conditions. Equal amounts of cell lysates or media were resolved by SDS-PAGE and analyzed by immunoblotting with the indicated antibodies. Results are representative of three independent experiments. In FGFR4-G388 cells, octreotide and pasireotide treatment can activate pY-STAT3. In contrast, in FGFR-R388 cells, only pasireotide can activate pY-STAT3, resulting in reduction of GH. **b**. GH4 cells expressing FGFR4-G388 or FGFR4-R388 were grown in soft agar in the absence or presence of somatostatin analogs. Shown are the colony numbers (mean ± SD of measurements obtained from four independent experiments). Pasireotide (Pasi) is more effective than octreotide (Oct) in inhibiting the enhanced growth of cells expressing FGFR4-R388.

To compare the impact of the different FGFR4 isoforms on SSTRs we first ensured that the FGFR4-R388 transmembrane substitution does not alter SSTR expression levels in pituitary GH4 mammosomatotroph cells (data not shown). To examine differential impact on signaling mediated by the two clinically available SSAs, octreotide and pasireotide, we compared their effect on phosphorylation of STAT3 tyrosine (pY-STAT3) and STAT3 serine (pS-STAT3). These signaling targets were selected on the basis of their pivotal roles in hormone regulation and cell growth in pituitary cells [20, 40]. Cells expressing the wild-type FGFR4 (FGFR4-G388) showed comparable signaling responsiveness to both somatostatin analogs (Figure 1a, 1b). In contrast, FGFR4-R388-expressing cells were relatively less sensitive to octreotide reduction of pS-STAT3 and to GH inhibition (Figure 1a). Moreover, pasireotide was significantly more effective than octreotide in reducing colony formation in FGFR4-R388 compared to FGFR4-G388 or to control cells (p-value=0.0003 and 0.004, respectively) (Figure 1b).

### Pasireotide, but not octreotide, interrupts STAT3 serine phosphorylation and its mitochondrial translocation

**Figure 2:**
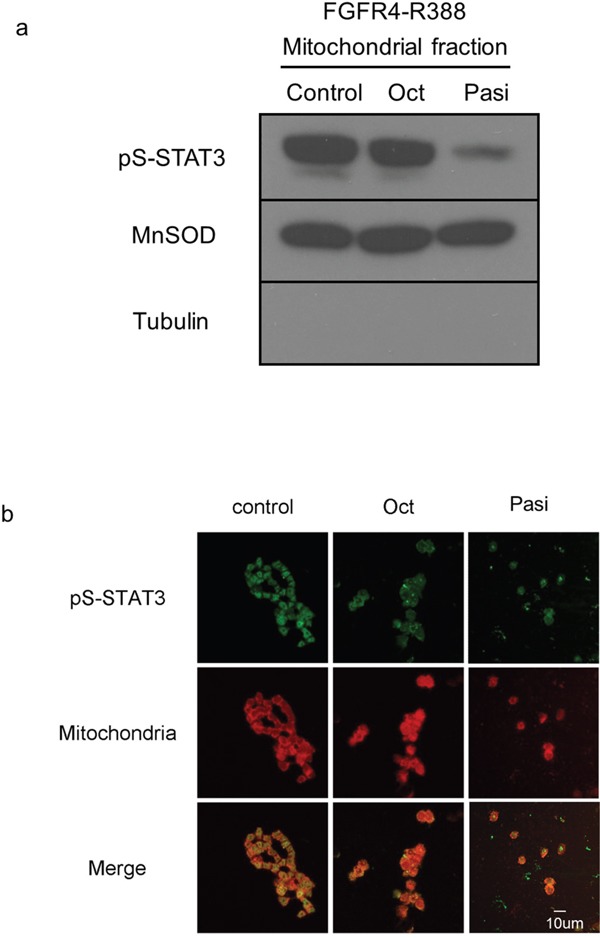
FGFR4-R388 responds to pasireotide, inducing serine-phosphorylated STAT3 translocation to mitochondria **a**. Equal amounts of fractionated proteins from pituitary GH4 cells expressing FGFR4-R388 treated with octreotide (Oct) or pasireotide (Pasi) were resolved by SDS-PAGE and immunoblotted for detection for pS-STAT3. MnSOD identifies mitochondrial enrichment and tubulin cytoplasmic exclusion. **b**. GH4 pituitary cells expressing FGFR4-R388 treated with octreotide (Oct) or pasireotide (Pasi), or their control were labeled with MitoTracker RED CMX Ros to visualize mitochondria and stained with anti-phospho-serine STAT3 followed by Alexa 488-conjugated secondary antibody. Pasireotide more effectively diminishes pS-STAT3 and its mitochondrial translocation than octreotide.

To examine the mechanisms underlying the differential effects of the somatostatin analogs on pituitary tumor cells, we investigated STAT3 serine phosphorylation. Mitochondrial fractionation studies revealed that pasireotide can reduce levels of pS-STAT3 in cells expressing FGFR4-R388 (Figure 2a). Additionally, immunofluorescence tracing studies corroborated the ability of pasireotide to reduce the mitochondrial pS-STAT3 in FGFR4-R388 cells (Figure 2b). Importantly, these actions were less evident with octreotide treatment (Figure 2a, 2b).

### FGFR4-R388 enhances mitochondrial functions through STAT3 in pituitary cells

**Figure 3:**
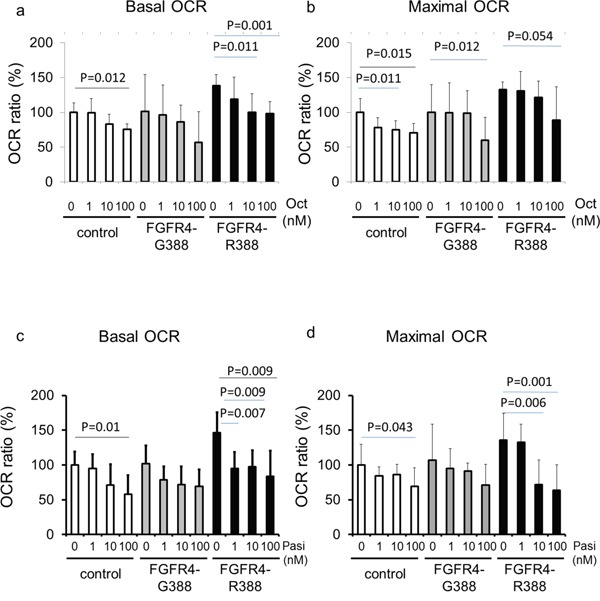
FGFR4-R388 activation of pS-STAT3 increases mitochondrial respiration in GH4 cells Equal numbers of GH4 cells expressing FGFR4-G388 or FGFR4-R388 were treated with octreotide (upper panels) or pasireotide (lower panels) for 24 hours. Basal oxygen consumption rate (OCR) (left panels) and maximal OCR (right panels) were measured using an extracellular flux analyzer as detailed under Materials & Methods. Relative OCRs shown represent the mean ± SD of 3 to 6 measurements obtained for each treatment dose for each of the indicated cell types. Statistically significant differences compared with control cells within each group by t-test are depicted.

To examine the functional relevance of the impact of SSAs on pS-STAT3 we performed mitochondrial measurements of basal and maximal oxygen consumption rates (OCRs) using proton flux analysis (Figure 3a-3d, Supplemental Figure 1a, 1b). These bioenergetic examinations revealed that pituitary GH4 cells expressing FGFR4-R388 display higher basal and maximal OCR levels than cells expressing the FGFR4-G388 isoform (Figure 3a-3d). Moreover, cells expressing FGFR4-R388 were sensitive to OCR reduction in response to pasireotide but not to octreotide. Specifically, pasireotide was significantly more effective than octreotide in reducing OCR in FGFR4-R388 compared to FGFR4-G388 isoform-expressing or control cells (p-value = 0.019 and 0.004, respectively). Additional data obtained in pituitary AtT20 corticotroph cells also revealed the increased ability of FGFR4-R388 to drive basal and maximal OCR in cells which were sensitive to pasireotide inhibition (p-value 0.005 and 0.001, respectively (Supplemental Figure 1a, 1b).

### Dysregulated STAT3 signaling is a mitochondrial target of pasireotide action in pituitary cells

**Figure 4:**
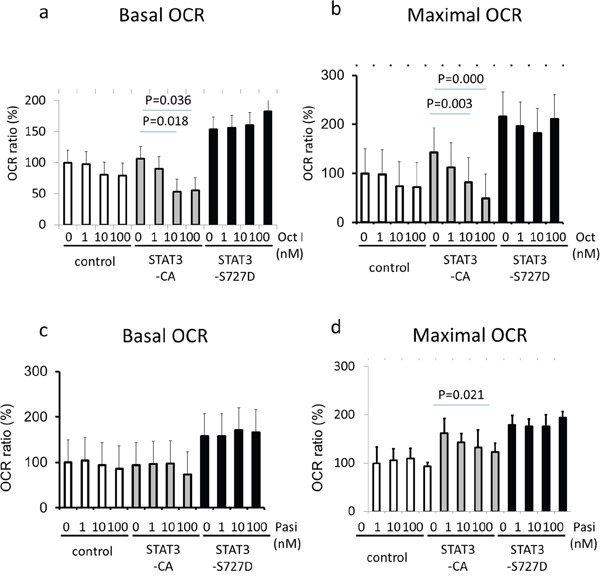
STAT3 serine activation induces mitochondrial respiratory resistance to pasireotide GH4 cells expressing empty vector (control), a constitutively tyrosine-active form of STAT3 (STAT3-CA), or a constitutively serine-active form of STAT3 (STAT3-S727D), were treated with octreotide (upper panels) or pasireotide (lower panels) for 24 hours. Basal oxygen consumption rate (OCR) (left panels) and maximal OCR (right panels) were measured using an extracellular flux analyzer as detailed under Materials & Methods. Relative OCRs shown represent the mean ± SD of 3 to 6 measurements obtained for each treatment dose for each of the indicated cell types. Statistically significant differences compared with control cells within each group by t-test are depicted.

To specifically determine if the higher pS-STAT3 levels endowed by the FGFR4-R388 variant allele are responsible for the observed differences in pituitary mitochondrial functions, we examined basal and maximal OCR in GH4 cells expressing STAT3 mutants (Figure 4). These studies showed that the constitutively active serine STAT3-S727D mutant can drive basal and maximal OCR to higher levels in these cells (Figure 4a, 4b). Interestingly, a tyrosine active STAT3-CA mutant could not recapitulate these actions as it was incapable of raising basal or maximal OCR levels in pituitary cells compared to their vector control cells (Figure 4a, 4b).

Importantly, pasireotide was significantly more effective than octreotide in reducing OCR in STAT3-S727D compared to STAT3-CA-expressing or control cells (p-value<0.0001 and =0.031, respectively).

### Pasireotide modulates STAT3 signaling through SSTR5 in pituitary cells

**Figure 5:**
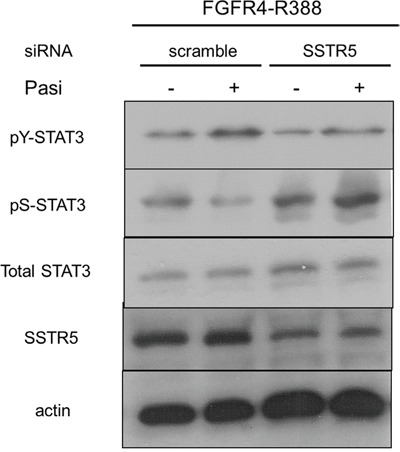
Pasireotide modulates STAT3 phosphorylation through SSTR5 in pituitary GH4 cells GH4 cells expressing FGFR4-R388 were transfected with siRNA oligonucleotides, either scrambled sequences or directed at SSTR5. Western blotting reveals that SSTR5 reduction abrogates the pasireotide-induced reduction of pS-STAT3 and induction of pY-STAT3.

One of the main recognized differences between octreotide and pasireotide is the higher affinity for the somatostatin SSTR5 receptor of pasireotide. We therefore determined if SSTR5 signaling is responsible for the differences we identified in FGFR4-R388 cells. In this regard, SSTR5 down-regulation resulted in increased pS-STAT3 and abolished the impact of pasireotide action on this STAT3 modification (Figure 5).

### Pasireotide targets phosphatase 2A to reduce STAT3 serine activation in pituitary cells

**Figure 6:**
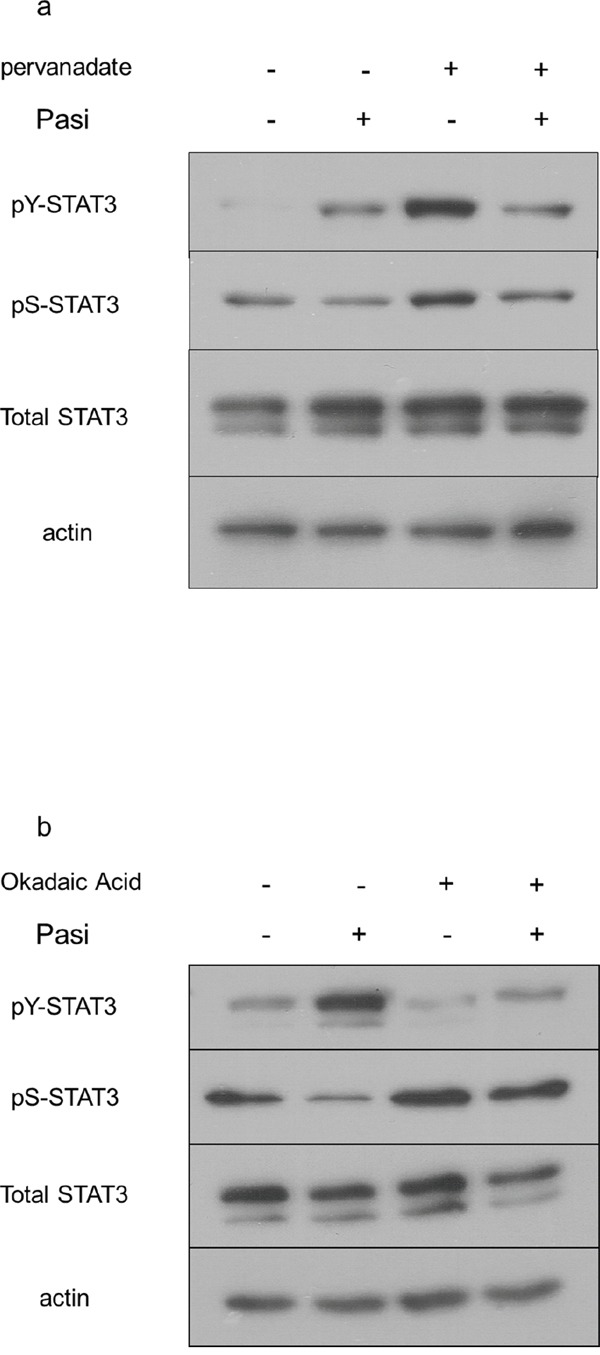
Pasireotide targets the phosphatase PP2A to diminish STAT3 signaling in GH4 cells GH4 cells expressing FGFR4-R388 were pretreated with the indicated phosphatase inhibitors followed by treatment with pasireotide (Pasi) as detailed under Materials & Methods. **a**. Pervanadate diminishes the effects of pasireotide on STAT3 signaling in GH4 cells. **b**. Okadaic acid interrupts the effect of pasireotide on STAT3 signaling in GH4 cells.

To examine the downstream targets of SSTR5-mediated pasireotide signaling which can be implicated in the differential impact on STAT3 phosphorylation, we tested the ability of phosphatase inhibitors to rescue these actions. To this end, the non-selective phosphatase inhibitor pervanadate diminished the impact of pasireotide on STAT3 serine dephosphorylation (Figure 6a). Additionally, the more selective phosphatase 2A inhibitor, okadaic acid, effectively diminished STAT3 dephosphorylation mediated by pasireotide, providing evidence for involvement of this phosphatase in promoting this drug's action (Figure 6b). In contrast, a phosphatase 2B inhibitor, cyclosporine A, did not alter STAT3 dephosphorylation by pasireotide (data not shown).

### Primary pituitary cells derived from FGFR4-KI mice show resistance to octreotide

**Figure 7:**
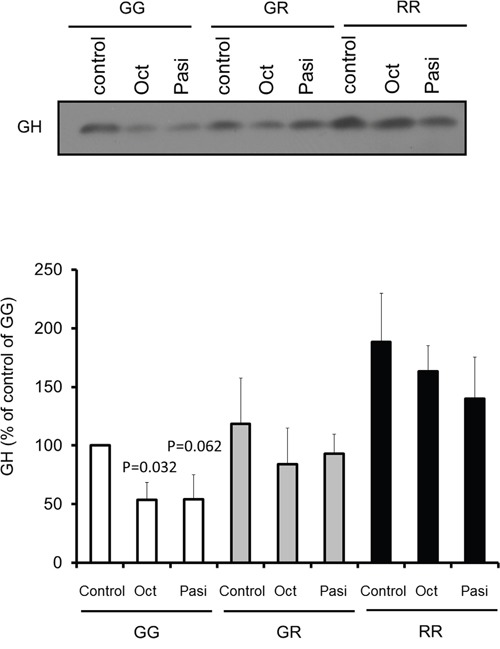
Impact of somatostatin analogs on primary pituitary cell signaling Equal numbers of primary pituitary cells from FGFR4-WT (GG) or FGFR4-R385 knock-in (heterozygous (GR) and homozygous (RR)) mice were plated on a 96 well plate, and treated with octreotide (Oct) or pasireotide (Pasi) under serum-free defined conditions. Equal volumes, 20 μl, of conditioned media were resolved by SDS-PAGE and analyzed by immunoblotting with the indicated antibodies for detection of hormone secretion. Densitometric ratios from three separate experiments are shown. Statistically significant differences compared with within each cell type are depicted.

To further study the impact of the FGFR4 SNP on response to somatostatin analogs we examined knock-in (KI) mice carrying the mouse homologue of the polymorphism, FGFR4-R385. Importantly, introduction of this SNP does not alter FGFR4 expression levels [39, 40]. Specifically, we tested the impact on GH secretion from primary pituitary cells from FGFR4-R385 KI mice mediated by octreotide and pasireotide. Primary pituitary cells derived from WT mice responded to both somatostatin analogs (Figure 7). In contrast, pituitary cells derived from mice carrying the FGFR4-R385 allele revealed diminished sensitivity to octreotide with attenuated GH reduction (Figure 7).

## DISCUSSION

The FGFR4-R388 SNP is known to be associated with accelerated cancer progression and treatment resistance [11–19]. The two FGFR4 isotypes have divergent signaling properties in different tissues as demonstrated in breast [18], pancreas [19, 42], pituitary mammosomatotroph cells [40] and corticotroph cells [20]. The FGFR4-R388 allele, which is associated with larger pituitary tumor size in patients with acromegaly, modulates STAT3 phosphorylation status, resulting in altered hormone regulation and enhanced cell proliferation in pituitary cells [20, 40]. Here we show that this transmembrane variant can mediate signaling that yields therapeutic targets of relevance to the actions of different SSAs. These differences can be noted when comparing effectiveness in reducing hormone production and cell proliferation and begin to explain the recognized differences in clinical efficacy of these compounds [43, 44].

SSAs activate SSTRs expressed on the cell surface, reduce hormone production and inhibit cell proliferation. Octreotide, which is used to treat patients with acromegaly or neuroendocrine tumors, has higher affinity for SSTR2. In contrast pasireotide has higher affinity for SSTR5. A proportion of acromegalic patients show resistance to octreotide, resulting in failure of IGF-1 normalization [45]. The predictive factors of responsiveness to SSAs have been examined, and include clinical features of patients, pathological features of GH-producing adenomas, and expression of SSTRs. We previously reported that the FGFR4-R388 allele modulates GH levels and is associated with larger pituitary tumor size in patients with acromegaly; STAT3 tyrosine phosphorylation is an inhibitory signal of GH secretion in pituitary cells and phosphorylated STAT3 serine in mitochondria plays a crucial role in cell proliferation [40].

**Figure 8:**
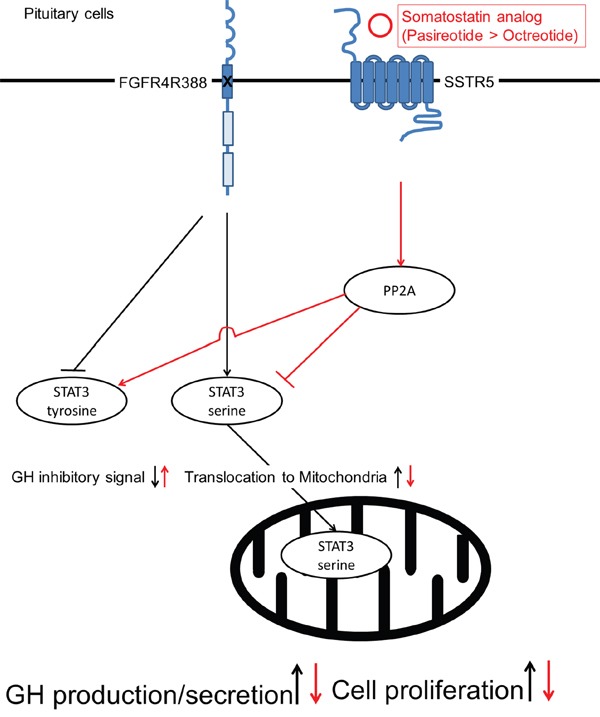
A proposed model for the mechanisms of pasireotide action on pituitary tumor cells Pasireotide can alter STAT3 phosphorylation through PP2A in pituitary cells carrying the FGFR4-R388 SNP more effectively than octreotide. The pasireotide-mediated increase in pY-STAT3 facilitates feedback inhibition resulting in GH suppression. Moreover, pasireotide-mediated STAT3 serine dephosphorylation reduces its mitochondrial translocation thereby limiting a critical signal for cellular growth and survival.

Our current data show that the FGFR4-R388 polymorphic variant leads to resistance to octreotide but maintains responsiveness to pasireotide. In contrast, the FGFR4-G388 form does not show resistance to octreotide or pasireotide. As the two FGFR4 isotypes do not alter the expression levels of SSTR2 or SSTR5, the differential STAT3 signaling may explain the mechanisms of resistance to octreotide in FGFR4-R388 cells (Figure 8).

Mitochondrial STAT3 has been shown to alter this organelle's functions including the electron transport chain, the mitochondrial permeability transition, ATP production, ROS production, transformation and cellular growth [46]. The FGFR4 SNP can alter STAT3 phosphorylation status in pituitary cells, thereby playing a critical role in hormone regulation and cell proliferation [20, 40]. Although STAT3 does not contain a mitochondrial targeting sequence, our data show that FGFR4-R388 induces enhanced mitochondrial respiration by phosphorylated STAT3 serine translocation to mitochondria in pituitary cells. Generally, proteins without mitochondrial targeting sequence utilize chaperone molecule(s) to translocate to mitochondria. Recently, the activated STAT3 serine was reported to increase its interaction with gene-associated with retinoid-interferon induced mortality-19 (GRIM-19), resulting in accumulation in the mitochondria [47, 48]. Loss of GRIM-19 reduces tumor cells respiration [49] supporting the importance of impaired STAT3 serine translocation into the mitochondria. Thus, it remains to be shown whether GRIM19 or other chaperones are directly involved in SSA-mediated control over pS-STAT3 and its mitochondrial residence. Nevertheless, the noted diminished STAT3 serine phosphorylation by pasireotide reduced its mitochondrial levels, thereby limiting a critical signal for cellular growth. Consistent with the impact of pS-STAT3, our bioenergetic studies corroborated the ability of FGFR4-R388 to augment basal and maximal mitochondrial respiration of pituitary tumor cells. Given the importance of oxidative phosphorylation on tumor cell survival [50–52], our findings uncover mitochondrial respiration as a novel site of SSA therapeutic action.

The pharmacologic actions of SSAs are now well-recognized to be mediated, at least in part, through phosphatase pathways [45]. Somatostatin can increase tyrosine phosphatases and serine/threonine phosphatase activity in human non-functioning pituitary tumors, GH-producing pituitary adenoma cells, and rat GH4 cells [53]. Okadaic acid, a serine/threonine phosphatase inhibitor, was shown to decrease phosphatase activity in GH4 cells, resulting in enhanced phosphorylation of RB protein and reduction of cell proliferation [54]. In the current study, okadaic acid inhibited STAT3 serine dephosphorylation induced by pasireotide (Figure 6) suggesting that this analog can act through phosphatase 2A in pituitary tumor cells carrying the FGFR4-R388 SNP. Further, we propose that SSA-mediated increase in pY-STAT3 facilitates GH feedback inhibition, providing a hormone control mechanism distinct from that required for cellular growth inhibition.

In summary, we show that pasireotide can modulate STAT3 phosphorylation through PP2A in pituitary cells carrying the FGFR4-R388 allele more effectively than octreotide. The diminished mitochondrial STAT3 signal serves to limit a critical signal for cellular growth and survival (Figure 8). Moreover, the ability of these analogs to increase pY-STAT3 is necessary to facilitate feedback hormone inhibition. These findings suggest that FGFR4 polymorphic isoforms mediate signaling that yields therapeutic targets of relevance to the distinct actions of clinically-available SSAs. The extent to which other genetic variants can be shown to selectively alter pituitary tumor signaling will form the basis for guiding pharmacogenetic decision-making therapeutic paradigms.

## MATERIALS AND METHODS

### Cell lines and cultures

As there are no human-derived hormone-producing pituitary cell lines, we used rat pituitary GH4 mammosomatotroph cells which were propagated in Ham F10 medium 12.5% horse and 2.5% fetal bovine serum (FBS; Sigma, Oakville, ON), 2 mM glutamine, 100 IU/ml penicillin, and 100 μg/ml streptomycin (37°C, 95% humidity, 5% CO_2_ atmosphere incubation). We developed GH4 cells stably expressing human prototypic FGFR4-G388 or the polymorphic form FGFR4-R388, which were selected using neomycin (G418) at a concentration of 0.7 μg/ml as previously reported [40], as well as mouse pituitary AtT20 corticotroph cells stably expressing FGFR4-G388 or FGFR4-R388, propagated in DMEM medium 10% FBS, 1mM sodium pyruvate, 100 IU/ml penicillin, 100 μg/ml streptomycin, 0.2μg/ml G418 (37°C, 95% humidity, 5% CO_2_ atmosphere incubation). A minimum of 3 clones of each isoform were used for further analyses in each of the cell types examined as described previously [40]. Mouse primary pituitaries were obtained from homozygous FGFR4-G385 or FGFGR4-R385 knock-in mice. Cells were dispersed for primary culture by mechanical agitation and enzymatic digestion by using 1 mg/ml of collagenase for 30 min at 37°C. Cells were pelleted by centrifugation at 1200 rpm for 5 min and re-suspended in serum-free DMEM containing 3 μg/ml putrescine, 1 x10^-6^ M hydrocortisone, 1 × 10^-11^ M tri-iodothyronine T3, 0.01 mg/ml insulin, transferring, and 0.375% albumin bovine factor V. Cells were plated on 0.01% poly-L-lysine-treated 96 well plate.

### siRNA knock-down

Oligonucleotides complementary to the gene of interest were synthesized by Shanghai Gene Pharma (Shanghai, China) and introduced by transfection using lipofectamine 2000 (Fisher Scientific, Ottawa). Scrambled sequences of equal length were used as controls.

### Cell treatments

Ligand stimulations were performed on cells grown in 100 mm plate (4 × 10^6^ cells/plate), pre-incubated as indicated for 1hr or 24 hrs in serum-free defined medium (3 μg/ml putrescine, 10^-6^ M hydrocortisone, 10^-11^ M tri-iodothyronine T3, and 0.375% albumin bovine factor V). For phosphatase inhibition, cells were serum deprived overnight and subsequently treated with pervanadate (0.1-100 μM) for 15 min. For PP2B inhibition we used cyclosporine A (Sigma, 10 μg/ml) for 1 hr. For PP2A inhibition, we used okadaic acid (Santa Cruz, Santa Cruz, CA) for 1 hour. Treatments with octreotide (Novartis, 1-100 nM), or pasireotide (Novartis, 1-100 nM), were based on earlier dose and time course studies ranging from 5 minutes up to 24 hrs.

### Mitochondrial isolation

Mitochondrial isolation was performed in accordance to the manufacturers’ protocol (Qproteome Mitochondria isolation kit (Qiagen) and as previously described [40]. Isolated fractions were analyzed by Western blotting to detect the phosphorylated STAT3 serine and MnSOD mitochondrial marker. Effective exclusion of contaminating cytoplasm was confirmed by detection of tubulin.

### Western blotting and antibodies

Cells were lysed in lysis buffer (0.5% sodium deoxycholate, 0.1% sodium dodecyl sulfate, 1% Nonidet P-40 and 1x PBS) containing proteinase inhibitors (100 μg/ml phenylmethylsulfonyl fluoride (PMSF), 13.8 μg/ml aprotinin (Sigma), and 1mM sodium orthovanadate (Sigma). Total cell lysates were incubated on ice for 30 mins, followed by micro-centrifugation at 10,000 g for 10 min at 4 °C. Protein concentrations of the supernatants were determined by Bio-Rad method. Equal amounts of protein (50 μg) were mixed with 5X SDS sample buffer, boiled for 5 mins and separated by 8, 10, or 12% sodium dodecyl sulfate (SDS)-polyacrylamide gel electrophoresis, and transferred onto PVDF membranes (0.45 μm, Millipore, US). Intracellular and secreted hormones were determined using the following antibodies: polyclonal antisera to PRL or GH [donated by the National Hormone and Pituitary Program (NHPP), National Institute of Diabetes and Digestive and Human Development, Bethesdsa, MD]. Immunoblotting was performed using anti-somatostatin receptor 5 (Abcam, Cambridge, MA), anti-pY-STAT3 (Y705, 1:1000), pS-STAT3 (S727, 1:1000), total STAT3 (1:2500), and anti-Tubulin (1:1000) which were purchased from Cell Signaling (Pickering, ON). Loading was also monitored by detection of actin (1:5000, Sigma). Non-specific binding was blocked with 5% nonfat milk in 1x TBST (Tris-buffered saline with 0.1% Tween-20). After washing for 3 × 10 mins in 1x TBST, blots were exposed to the secondary antibody (anti-rabbit or mouse IgG-HRP, Santa Cruz) at a dilution of 1:2000 and were visualized using ECL chemiluminescence detection system (Amersham, U.K.).

### Measurement of cellular oxygen consumption

Oxygen consumption rate (OCR) was measured in real-time, in an XFe96 Extracellular Flux Analyzer (Seahorse Bioscience, Billerica, MA). GH4 cells stably expressing the FGFR4-G388, the FGFR4-R388, or STAT3 mutants were seeded in XF^e^96-well plates (50,000 cells per well in 100 μl), and incubated with or without treatment with octreotide (1-100 nM) or pasireotide (1-100 nM) overnight at 37°C, 5% CO2. The XF^e^96 sensor cartridge was hydrated with 200 μl calibration buffer per well overnight at 37°C. The sensor cartridge was loaded with assay media (ports A, B and C) to measure the bioenergetic profile with or without oligomycin (1 μM, port A), carbonyl cyanide 4-(trifluoromethoxy) phenylhydrazone (FCCP, 0.5 μM, port B), and rotenone and antimycin A (1 μM, port C). Cells were washed once with pre-warmed serum-free un-buffered assay medium. 162 μl/well of assay medium was added for measurement of basal OCR. Once the sensor cartridge was equilibrated, the calibration plate was replaced with the assay plate. To determine the bioenergetic profile, measurements were taken every 3 minutes.

### Immunofluorescence detection of phospho-STAT3 serine

Cells were grown in 2 chamber slides and pre-incubated in serum-free defined medium for 16 hrs. Cells were treated with or without somatostatin analogs (octreotide 100 nM or pasireotide 100 nM) for 24 hrs. Cells were incubated with MitoTracker Red CMXRos (Invitrogen) at 37°C for 20 minutes, washed twice with PBS, fixed with 4% formaldehyde/PBS for 10 minutes, and washed three times with PBS. Cells were permeabilized for 10 minutes in PBS with 0.2% Triton X-100 and blocked for 30 minutes with PBS containing 5% FBS. Cells were first incubated with rabbit anti- pS-STAT3 antibody (1:100) for 30 minutes at room temperature, washed three times with PBS, subsequently incubated with anti-rabbit IgG Alexa Fluor 488 for 30 minutes at room temperature, and washed three times with PBS. Coverslips were mounted in Fluoromount-G purchased from Electron Microscopy Sciences (Hatfield, PA) on glass slides. Cells were examined with confocal microscope (Zeiss LSM 700) equipped with a 63× oil-immersion objective lens and filters optimized for double-label experiments. Images were examined using the LSM IMAGE browser.

### FGFR4-R385 knock-in mice

FGFR4-R385 knock-in (KI) mice were generated as described previously [40]. Mice were maintained on a pure C57BL/6 background. Genotyping was performed by PCR of genomic tail-DNA [41].

### Ethics statement

The care of animals was approved by the Institutional Animal Care facilities.

### Statistical analysis

Data are presented as mean ± standard deviation (SD). Differences between control and treatments within the same cell type were assessed by *t*-test. For each dataset, linear regression models were fitted to colony counts, basal OCR or maximal OCR. The model selection process was the same for every dataset. First, a linear regression model with three way interaction was fitted. Backward selection technique with likelihood ratio test was used to obtain the final model. To test for differences in drug effects between different cell types, the interaction between drug and cell type was the main effect of interest.

## SUPPLEMENTARY MATERIALS FIGURES


