# Supplementary material for: FGFR4 polymorphic alleles modulate mitochondrial respiration: A novel target for somatostatin analog action in pituitary tumors
Source: Oncotarget. 2016 Dec 9;8(2):3481–94. doi: 10.18632/oncotarget.13843 (PMC5356897; doi:10.18632/oncotarget.13843)
Supplement: Supplementary file 1 [file oncotarget-08-3481-s001.pdf]

FGFR4 polymorphic alleles modulate mitochondrial respiration: A novel target for somatostatin analog action in pituitary tumors

SUPPLEMENTARY FIGURE

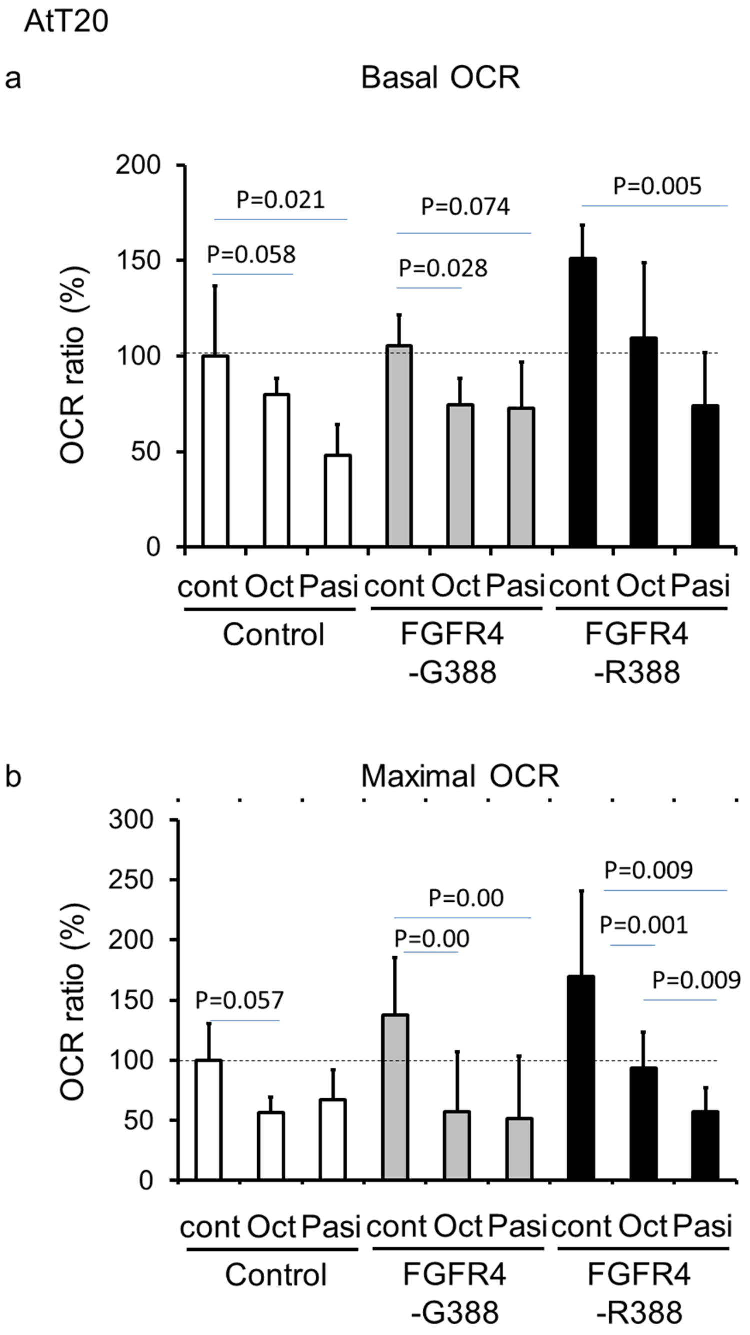

Supplementary Figure S1: FGFR4-R388 enhances mitochondrial respiration in mouse corticotroph AtT20 cells. Equal numbers of pituitary AtT20 cells expressing FGFR4-G388, FGFR4-R388, or their controls were treated with or without somatostatin analog (100 nM octreotide or 100 nM pasireotide) for 24 hours. Basal **a.** and maximal **b.** oxygen consumption rates (OCRs) were measured using an extracellular flux analyzer as detailed under Materials & Methods. Statistically significant differences compared within each cell type are depicted.
